# Supplementary material for: Seminoma and Embryonal Carcinoma Footprints Identified by Analysis of Integrated Genome-Wide Epigenetic and Expression Profiles of Germ Cell Cancer Cell Lines
Source: PLoS One. 2014 Jun 2;9(6):e98330. doi: 10.1371/journal.pone.0098330 (PMC4041891; doi:10.1371/journal.pone.0098330)
Supplement: File S1 — ZIP file containing DMRforPairs output for significant regions. Please start from the html files. (ZIP) [file pone.0098330.s008.zip › figures/27674.pdf]

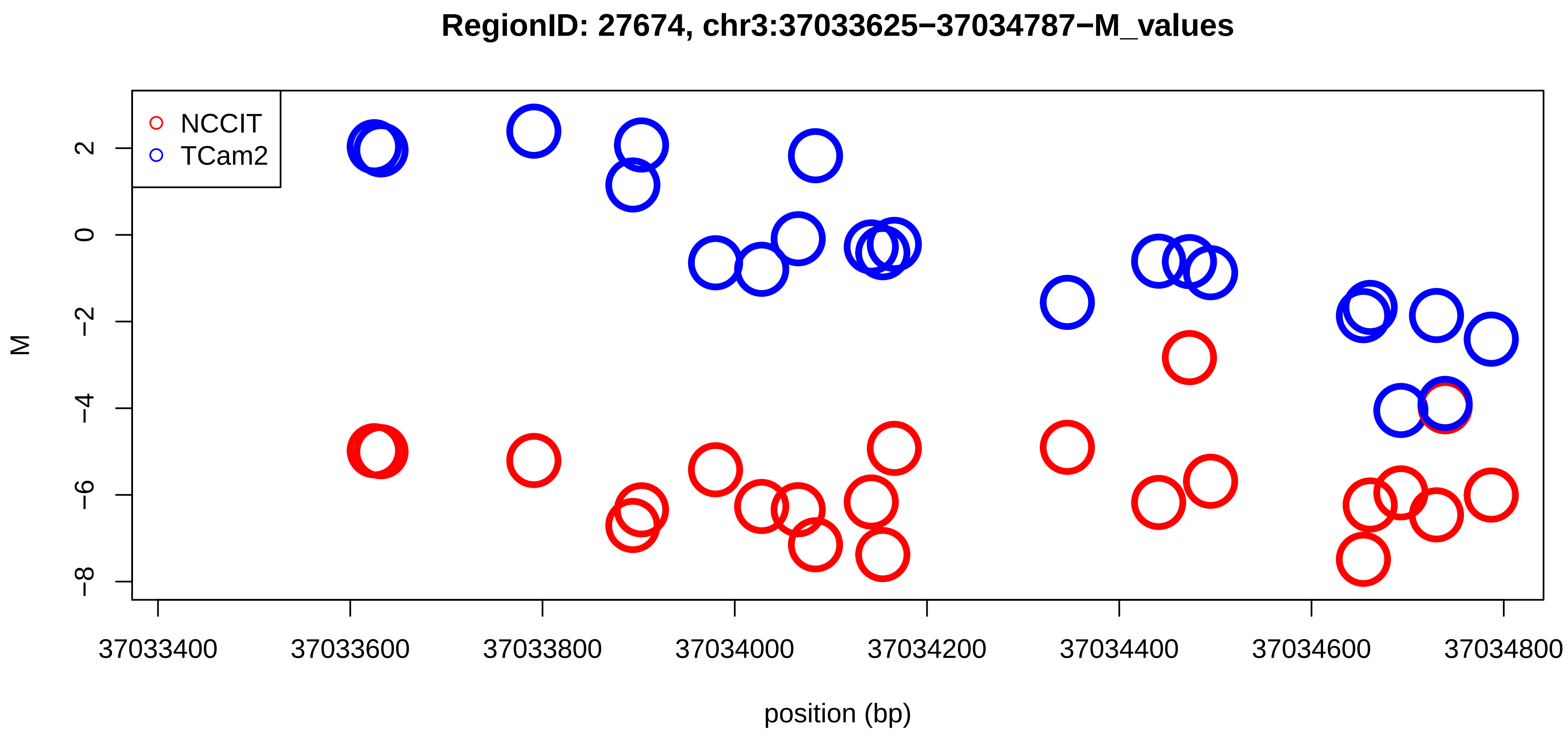

RegionID: 27674, chr3:37033625−37034787−M_values
NCCIT
TCam2
M
2
0
−2
−4
−6
−8
37033400
37033600
37033800
37034000
37034200
37034400
37034600
37034800
position (bp)

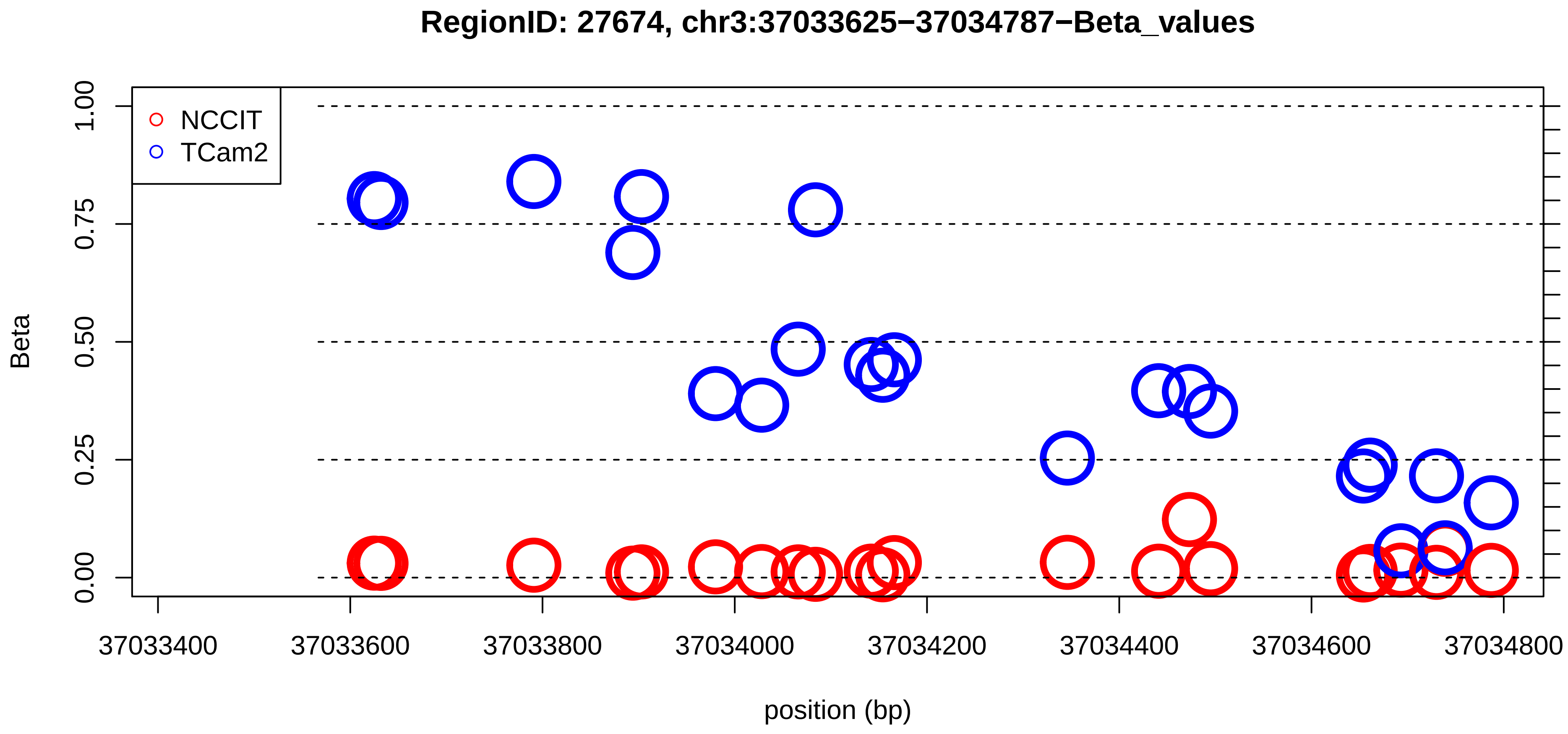

RegionID: 27674, chr3:37033625−37034787−Beta_values
NCCIT
TCam2
Beta
1.00
0.75
0.50
0.25
0.00
37033400
37033600
37033800
37034000
37034200
37034400
37034600
37034800
position (bp)

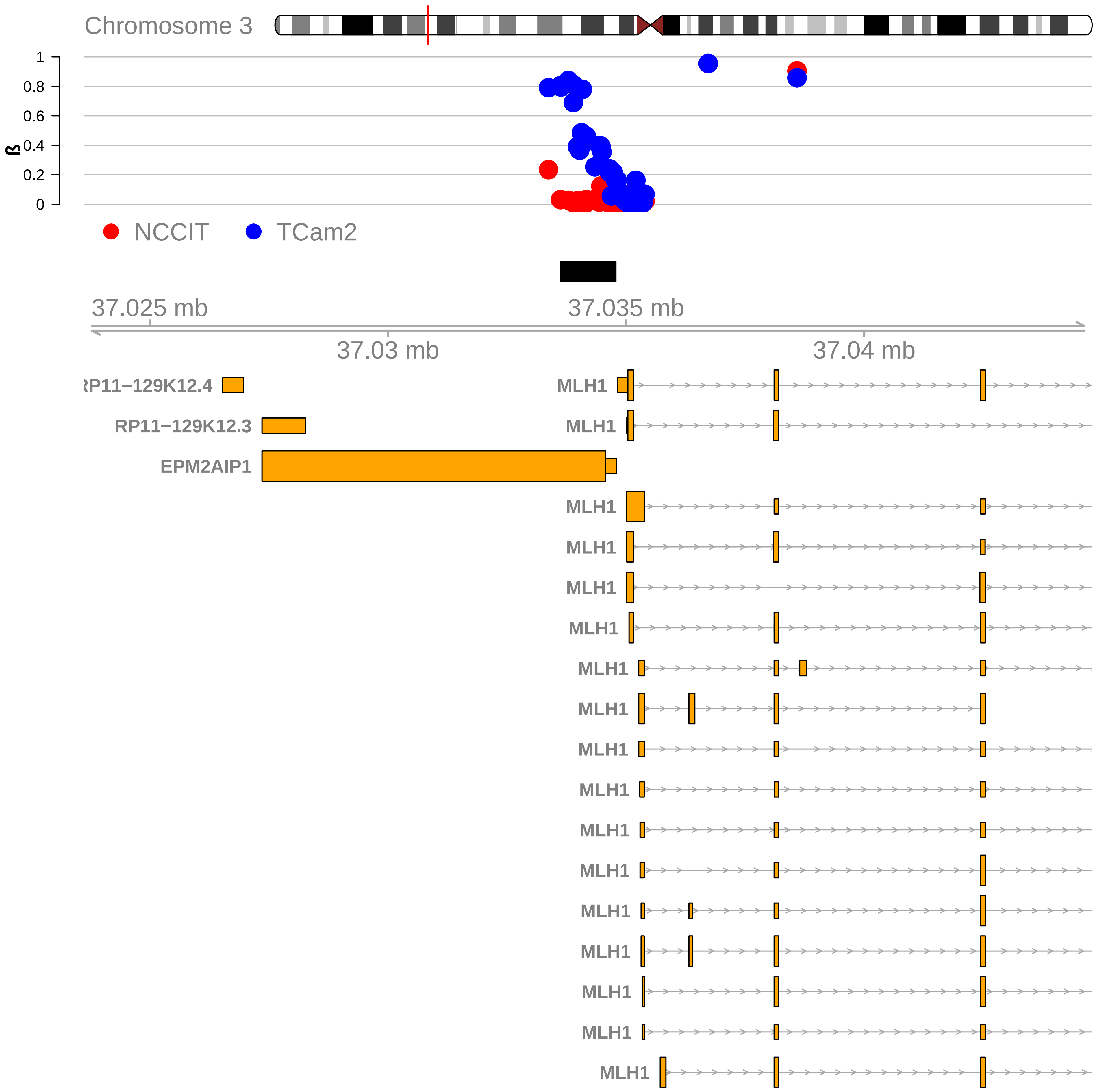

Chromosome 3
1
0.8
0.6
0.4
0.2
0
β
NCCIT
TCam2
37.025 mb
37.035 mb
37.03 mb
37.04 mb
RP11−129K12.4
RP11−129K12.3
EPM2AIP1
MLH1
